# Temperature and Water Availability During Maturation Affect the Cytokinins and Auxins Profile of Radiata Pine Somatic Embryos

**DOI:** 10.3389/fpls.2018.01898

**Published:** 2018-12-20

**Authors:** Paloma Moncaleán, Olatz García-Mendiguren, Ondrej Novák, Miroslav Strnad, Tomás Goicoa, María D. Ugarte, Itziar A. Montalbán

**Affiliations:** ^1^Department of Forestry Science, NEIKER-Tecnalia, Arkaute, Spain; ^2^Laboratory of Growth Regulators, Centre of the Region Haná for Biotechnological and Agricultural Research, Faculty of Science, Institute of Experimental Botany CAS, Palacký University Olomouc, Olomouc, Czechia; ^3^Department of Statistics, Computer Science and Mathematics, Universidad Pública de Navarra, Pamplona, Spain

**Keywords:** cytokinins, embryonal masses, embryogenic cell lines, indol-3-acetic acid, *Pinus radiata*, somatic embryogenesis

## Abstract

Somatic embryogenesis (SE) provides us a potent biotechnological tool to manipulate the physical and chemical conditions (water availability) along the process and to study their effect in the final success in terms of quantity of somatic embryos produced. In the last years, our research team has been focused on the study of different aspects of the SE in *Pinus* spp. One of the main aspects affecting SE is the composition of culture media; in this sense, phytohormones play one of the most crucial roles in this propagation system. Many studies in conifers have shown that different stages of SE and somatic embryo development are correlated with distinct endogenous phytohormone profiles under the stress conditions needed for the process (i.e., cytokinins play a regulatory role in stress signaling, which it is essential for radiata pine SE). Based on this knowledge, the aim of this study was to test the effect of different temperatures (18, 23, and 28°C) and gelling agent concentrations (8, 9, and 10 gL^-1^) during the maturation stage of *Pinus radiata* SE in maturation and germination rates. Parallel, phytohormone profile of somatic embryos developed was evaluated. In this sense, the highest gellan gum concentration led to significantly lower water availability. At this gellan gum concentration and 23°C a significantly higher number of somatic embryos was obtained and the overall success of the process increased with respect to other treatments assayed. The somatic embryos produced in these conditions showed the highest concentration of iP-type cytokinins and total ribosides. Although, the different conditions applied during maturation of somatic embryos led to different hormonal profiles, they did not affect the *ex vitro* survival of the resulting somatic plants, where no significant differences were observed.

## Introduction

The use of vegetative propagation in forestry is the fastest, most flexible and effective way to produce enough genetically improved material to meet future wood demands (Lelu-Walter et al., 2013). Somatic embryogenesis (SE) is currently considered one of the successful biotechnological techniques for mass propagation, enabling multi-varietal forestry (Park et al., 2016) and for cryopreservation of embryonal masses (EMs) from elite genotypes (Corredoira et al., 2006).

In the last years, several improvements in different aspects of *Pinus* spp. SE process, such as initiation (Montalbán et al., 2012), maturation (Montalbán et al., 2010) and germination (Montalbán and Moncaleán, 2018) have been carried out in our laboratory. Traditionally, the adjustments have been focused on modifications of basal medium components (Salajova and Salaj, 2005; Zhang et al., 2007), exposure to different types and concentrations of exogenous plant growth regulators (Choudhury et al., 2008) and the use of different initial explants (Hargreaves et al., 2017).

One of the main bottlenecks of the SE in conifers is the progression from immature embryogenic cultures into mature cotyledonary embryos able to develop well-growing plants (Harvengt, 2005). Several external stimuli, such as plant growth regulators (Ayil-Gutiérrez et al., 2013), osmotic agents (Kikuchi et al., 2006), nutritional components (Pěnčík et al., 2015), amongst others, have been recognized as essential factors in determining both the hormone biosynthesis and the developmental fate of explant cells (Grzyb et al., 2017). In conifers, maturation is stimulated by reduction of water availability by means of raising the osmoticum of the medium (Montalbán et al., 2010) or the concentration of gelling agent (Klimaszewska and Smith, 1997). Modifying gellan gum concentration in maturation media has been studied in a few species of angiosperms (Márquez-Martín et al., 2011) and gymnosperms (Morel et al., 2014); these authors reported an improvement in the development and maturation of somatic embryos (Se) of *P. pinaster* by increasing gellan gum concentration, pointing out that the effect of gellan gum concentration on plant response is provoked by changes of water availability in the culture medium. In our laboratory, we demonstrated in previous studies that the agar concentration and the temperature had a significant effect on initiation step of radiata pine SE; and in the case of temperature, in the subsequent phases of the process (García-Mendiguren et al., 2016). As far as we know, the effect of different temperatures in maturation step has not been tested in *Pinus* spp., only in some angiosperms (Wang et al., 2014).

The active cytokinin (CK) pool is regulated during development by biosynthesis, uptake from extracellular sources, metabolic interconversions, inactivation, degradation, and transport (Kakimoto, 2003). The relative abundance of different CKs can vary greatly between plant species, tissues and developmental stages, and depends on the environmental conditions (Frébort et al., 2011). Some studies have examined the effect of varying the culture atmosphere on endogenous CK levels after organogenesis in angiosperms (Jiménez and Bangerth, 2001; Moncaleán et al., 2003) and gymnosperms (Moncaleán et al., 2005; Montalbán et al., 2012). On the other hand, the role of indole-3- acetic acid (IAA) and CKs in the SE process have been analyzed in cotton (Zeng et al., 2007; Jin et al., 2014) and wheat (Hess and Carman, 1998; Jiménez and Bangerth, 2001) and fern (Grzyb et al., 2017). However, up to know a deep analysis of endogenous cytokinins and IAA concentration in SE process in pines has not been determined. In this sense, and as it has been described previously, IAA and other phytohormones have an important role in embryo differentiation events (Silveira et al., 2004; Vondrakova et al., 2018); thus, a better understanding of the phytohormone profile in somatic embryos under different environmental conditions could enable us to manipulate this stage of SE to increase its efficiency. Taking into account all the above mentioned studies, the main objective of this study was the evaluation of how the environment of cultures (gellan gum concentration and temperature) along maturation stage affects the success of radiata pine SE processes as well as to analyze its effect in CK and IAA endogenous levels.

## Materials and Methods

### Maturation Experiment

#### Plant Material

**FIGURE 1:**
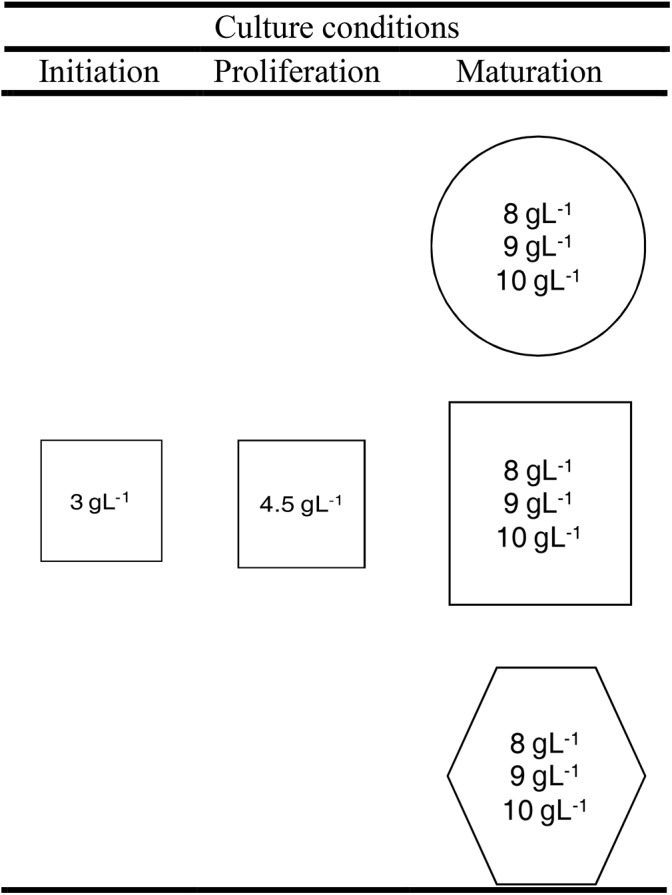
Scheme of the experimental design of maturation experiment. Cultures were stored at three different temperatures: 18°C (circle), 23°C (square), or 28°C (hexagon) and at three different agar concentrations (inside circles, squares, and hexagons).

Ten embryogenic cell lines (ECLs) from four open-pollinated *Pinus radiata* D. Don trees were selected for the experiments. The trees belonged to a seed orchard established by Neiker-Tecnalia in Deba (Spain; latitude: 43°16′59′N, longitude: 2°17′59′W, elevation: 50 m). The ECLs were initiated and proliferated in clumps in standard conditions following Montalbán et al. (2012) (Figure 1).

#### Maturation Procedure

The basal medium used in all phases of SE process was Embryo Development Medium (EDM, Walter et al., 2005). For initiation and proliferation 30 gL^-1^ sucrose, 4.5 μM 2,4-dichlorophenoxyacetic acid and 2.7 μM benzyladenine (BA) were added. As gelling agent, 3 gL^-1^ and 4.5 gL^-1^ Gelrite^®^were used for initiation and proliferation, respectively. The pH was adjusted to 5.7 prior to sterilization at 121°C for 20 min. After autoclaving, filter-sterilized solutions (pH 5.7) of the following amino acids (Walter et al., 2005) were added to partially cooled medium prior to dispensing into Petri dishes (90 mm × 20 mm): 550 mgL^-1^ L-glutamine, 525 mgL^-1^ L-asparagine, 175 mgL^-1^ L-arginine, 19.75 mgL^-1^ L-citrulline, 19 mgL^-1^ L-ornithine, 13.75 mgL^-1^ L-lysine, 10 mgL^-1^ L-alanine, and 8.75 mgL^-1^ L-proline. All chemical products were purchased from Duchefa.

**FIGURE 2:**
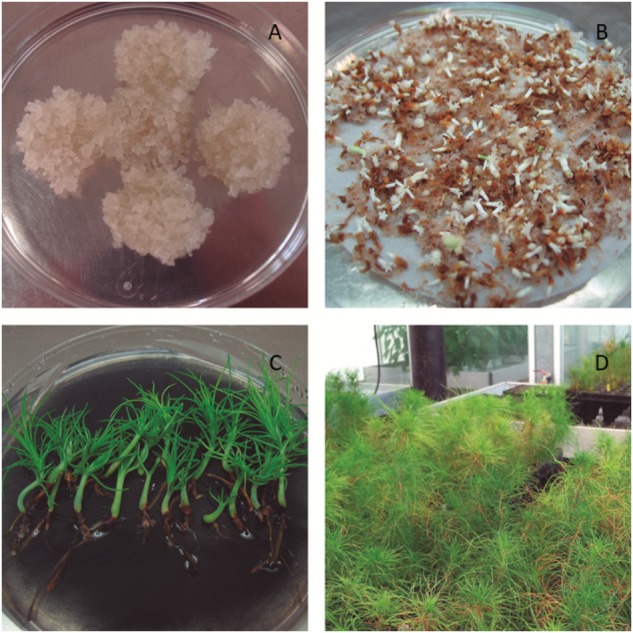
*Pinus radiata* embryonal masses **(A)**, mature somatic embryos **(B)**, germinated somatic planlets **(C)**, and acclimatized somatic plants **(D)**.

Three weeks before the start of maturation experiment, EMs (Figure 2A) were suspended in liquid growth regulators-free basal medium in 50 mL centrifuge tubes; then the suspension was poured onto a filter paper disk (Whatman No. 2) in a Büchner funnel. The filter papers with 250 mg of EMs each were laid on proliferation media. Maturation of ECLs was carried out following the protocol described by Montalbán et al. (2010). Briefly, EMs were suspended in liquid growth regulators-free basal medium and filtered as described above. The filter papers with 80 mg of EMs each were laid on maturation media. The basal media used were the same as described before supplemented 60 gL^-1^ sucrose and the amino acid mixture used for initiation and proliferation of the EMs. Also, 60 μM abscisic acid (ABA, purchased from Olchemim) was added.

In order to increase or reduce the water availability of the medium, the amount of Gelrite^®^in the maturation medium was increased (10 gL^-1^) or reduced (8 gL^-1^), respectively, respect to the standard conditions (9 gL^-1^). The cultures were kept under different temperatures, 5°C above (28°C) and below (18°C) the standard (23°C). Thus, nine different treatments were tested, carrying out the other SE steps under standard conditions (Figure 1). Summarizing, the whole experiment comprised nine different treatments, 10 ECLs and four replicates per ECL and maturation condition (a total of 360 maturation plates). All the cultures were kept in darkness.

#### Germination Procedure

After 15–18 weeks, Se were transferred to germination medium. This medium was half strength macronutrients LP (Quoirin and Lepoivre, 1977, modified by Aitken-Christie et al., 1988) supplemented with 2 gL^-1^ activated charcoal (AC, Sigma-Aldrich) and 9.5 gL^-1^ Difco Agar granulated (Becton & Dickinson). In those ECLs and maturation conditions producing Se, three to four Petri dishes, and 20 Se per Petri dish, with the embryonic root caps pointing downward, were titled vertically at an angle of approximately 45°, and cultured at 23 ± 1°C under 16 h photoperiod at 100 μmolm^-2^s^-1^ provided by cool white fluorescent tubes (TFL 58 W/33; Philips, France). Plantlets were subcultured to culture vessels with medium of the same composition after 6 weeks.

After a germination period of 14–16 weeks, plantlets were transferred to sterile peat:perlite (7:3, v/v) and acclimatized in a greenhouse with environmental control where the humidity was progressively decreased from 99 to 70% during the first month.

#### Water Availability Determination

The water availability of maturation media (EDM) under different maturation conditions (three gellan gum concentrations and three temperatures) was determined by weighing and placing one autoclaved filter paper disk (Whatman No. 2) on the surface of the medium following Klimaszewska et al. (2000). The Petri dishes were incubated under the same conditions as those used for maturation for 15 weeks. Then, the filter paper disks were weighed and the amount of water (g) absorbed was calculated. Four replicates per maturation condition were used.

### Extraction, Purification and Quantification of Endogenous Cytokinins

#### Plant Material

Somatic embryos (Figure 2B) obtained from EMs maturated at 18, 23, and 28°C in EDM medium solidified with 8, 9, and 10 gL^-1^ (Figure 1).

#### Quantitative Analysis of CKs and IAA by Liquid Chromatography-Single Quadrupole MS

The 41 CKs and auxins analyzed were the following: *cis*-Zeatin (cZ), *cis*-Zeatin riboside (cZR), *cis*-Zeatin *O*-glucoside (cZOG), *cis*-Zeatin-9-glucoside (cZ9G), *cis*-Zeatin riboside *O*-glucoside (cZROG), *cis*-Zeatin riboside-5′-monophosphate (cZMP), *trans*-Zeatin (tZ), *trans*-Zeatin riboside (tZR), *trans*-Zeatin *O*-glucoside (tZOG), *trans*-Zeatin-7-glucoside (tZ7G), *trans*-Zeatin-9-glucoside (tZ9G), *trans*-Zeatin riboside *O*-glucoside (tZROG), *trans*-Zeatin riboside-5′-monophosphate (tZMP), Dihydrozeatin (DHZ), Dihydrozeatin riboside (DHZR), Dihydrozeatin *O*-glucoside (DHZOG), Dihydrozeatin-7-glucoside (DHZ7G), Dihydrozeatin-9-glucoside (DHZ9G), Dihydrozeatin riboside *O*-glucoside (DHZROG), Dihydrozeatin riboside-5′-monophosphate (DHZMP), N^6^-Isopentenyladenine (iP), N^6^-Isopentenyladenosine (iPR), N^6^-Isopentenyladenine-7-glucoside (iP7G), N^6^-Isopentenyladenine-9-glucoside (iP9G), N^6^-Isopentenyladenosine-5′-monophosphate (iPMP), N^6^-Benzyladenine (BA), N^6^-Benzyladenosine (BAR), N^6^-Benzyladenine-9-glucoside (BA9G), N^6^-benzyladenosine-5′-monophosphate (BARMP), *ortho*-Topolin (oT), *ortho*-Topolin riboside (oTR), *ortho*-Topolin-9-glucoside (oT9G), *meta*-Topolin (mT), *meta*-Topolin riboside (mTR), *meta*-Topolin-9-glucoside (mT9G), *para*-Topolin (pT), *para*-Topolin riboside (pTR). Indol-3-acetic acid (IAA), 2-oxindole-3-acetic acid (oxIAA), IAA-aspartate (IAA-Asp), and IAA-glutamate (IAA-Glu).

Three replicates of 3 mg of Se were analyzed according to the protocol described by Svačinová et al. (2012) and Pěnčík et al. (2018) for cytokinin and auxin profiling, respectively, using miniaturized purification (pipette tip solid-phase extraction). Samples were extracted with the addition of stable isotope-labeled internal standards (each at 0.5 pmol per sample) and transferred after extraction onto Stage Tips and purified according to the aforementioned protocol, consisting of C18, SDB-RPS, and Cation-SR sorbents for cytokinins or C18 and SDB-XC sorbents for IAA and IAA conjugates. Eluates were collected into new clean microcentrifuge tubes and evaporated to dryness in a Speed-Vac concentrator and dissolved in 30 μl of mobile phase prior to UHPLC-MS/MS analyses.

Mass analysis was carried out using an Acquity UPLC^®^System (Waters, Milford, MA, United States), and a triple-quadrupole mass spectrometer Xevo^TM^ TQ-S MS (Waters MS Technologies, Manchester, United Kingdom). Quantification was achieved by monitoring protonated precursors and the appropriate product ions. Multiple reaction monitoring transitions as well as chromatographic and tandem mass spectrometry (MS) conditions were selected according to the method described by Novák et al. (2008, 2012). All MS data were processed using the MassLynx^TM^ software with TargetLynx^TM^ program (version 4.2. Waters, Milford, MA, United States), and compounds were quantified by standard isotope dilution analysis (Rittenberg and Foster, 1940).

### Data Collection and Statistical Analysis

The ECLs subjected to different maturation conditions that had produced Se were registered and the percentage of maturation and the number of Se per gram of EM (fresh weight) were calculated. The number of somatic embryos from different maturation conditions that had germinated was recorded and germination percentages were calculated (Figure 2C). A logistic regression and the corresponding analysis of deviance were conducted to evaluate the effect of temperature and gellan gum concentration on different stages of plant conversion for *P. radiata*.

Then, the success rate (plants able to be planted in *ex vitro* conditions) was evaluated in plantlets coming from ECLs cultured under different maturation treatments and it was calculated with respect to standard conditions (23°C and 9 gL^-1^ gellan gum, considered as 100% for maturation rate, number of Se per gram of EM and germination percentage).

After 14 weeks under *ex vitro* conditions (Figure 2D), the survival was calculated. To evaluate the effect of the maturation treatments, an analysis of variance (ANOVA) was carried out.

For water availability analyses, a two-way analysis of variance (ANOVA) was carried out to assess the effect of temperature and gellan gum concentration on the water availability of the media. When significant differences between the levels of the two variables were found, the Tukey HSD *post hoc* test was carried out to find out which levels were statistically different.

Data for the different CK types and metabolites, for IAA contents and the ratio of IAA: active CKs were evaluated using a two-way ANOVA to identify differences between treatments. Multiple comparisons were performed using Tukey’s *post hoc* test.

## Results

### Maturation Experiment

Detailed information on the statistical analyses performed is given as Supplementary Material.

**FIGURE 3:**
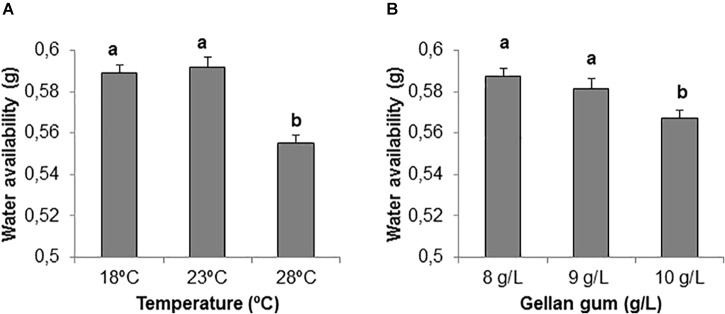
Water availability (g) on EDM medium **(A)** stored at three different temperatures (18, 23, and 28°C), **(B)** supplemented with three different gellan gum concentrations (8, 9, and 10 g L^-1^). Different letters show significant differences at a significance level of α = 0.05. Pairwise comparisons have been assessed with the Tukey HSD *post hoc* test.

Significant differences were found among the levels of gellan gum and temperature although the interaction between the tested variables was not significant. In this sense, the highest temperature analyzed (28°C) led to a significantly lower water availability than the other temperatures tested (Figure 3A). EDM medium supplemented with 10 gL^-1^ gelrite showed also significantly lower water availability than those supplemented with 8 or 9 gL^-1^ (Figure 3B).

**FIGURE 4:**
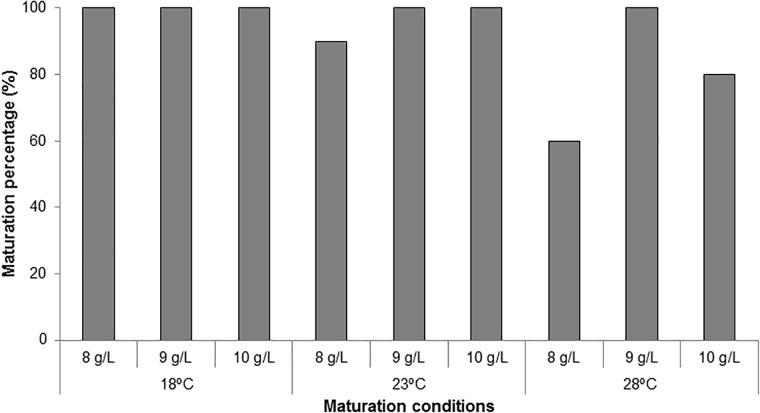
Maturation percentages (%) in *P. radiata* for embryogenic cell lines cultured at three different temperatures (18, 23, and 28°C) and three gellan gum concentrations (8, 9, and 10 g L^-1^).

Maturation percentages were 100% in all treatments except in ECLs cultured at 23 and 28°C in culture media solidified with 8 gL^-1^ gelrite (90 and 60%, respectively) and in those cultured at 28°C with 10 gL^-1^ gelrite (80%, Figure 4).

**FIGURE 5:**
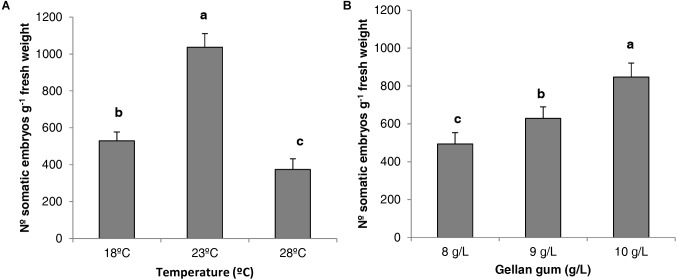
Number of *P. radiata* somatic embryos per gram of embryogenic tissue (fresh weight) maturated on EDM medium **(A)** at three different temperatures (18, 23, and 28°C), **(B)** supplemented with three different gellan gum concentrations (8, 9, and 10 g L^-1^). Different letters show significant differences at a significance level of α = 0.05. Pairwise comparisons have been assessed with the Tukey HSD *post hoc* test.

When the analysis of deviance for the number of *P. radiata* Se was evaluated, statistical differences were found for the different temperatures and gellan gum concentrations tested but no interaction between them was found. The Tukey HSD *post hoc* test revealed significant differences between the three temperatures tested; being 23 and 28°C the temperatures that produced the highest and the lowest number of Se (1036 and 374 Seg^-1^ EM, respectively, Figure 5A). Moreover, ECLs maturated in culture media with the lowest water availability (10 gL^-1^) produced a significantly higher number of somatic embryos (Figure 5B).

**FIGURE 6:**
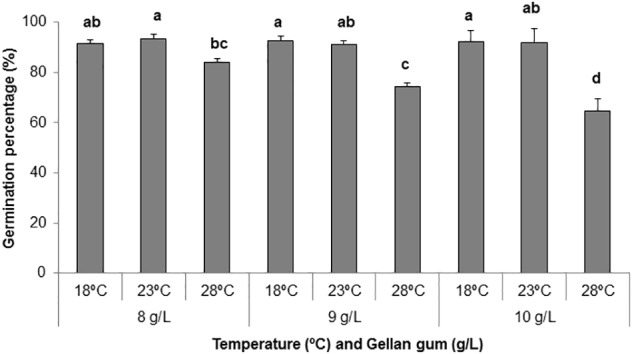
Germination percentages (%) of *P. radiata* somatic embryos maturated on EDM medium supplemented with three different gellan gum concentrations (8, 9, and 10 g L^-1^) and stored at three different temperatures (18, 23, and 28°C). Different letters show significant differences at a significance level of α = 0.05. Pairwise comparisons have been assessed with the Tukey HSD *post hoc* test.

Regarding germination percentage, significant interaction between temperature, and gellan gum concentration at maturation was observed. The highest germination rates, above 90%, were obtained when EMs were maturated at 18 and 23°C, whereas maturation at 28°C led to lower germination rates, particularly when it was combined with 9 or 10 gL^-1^ gellan gum (74 and 64%, respectively, Figure 6).

**FIGURE 7:**
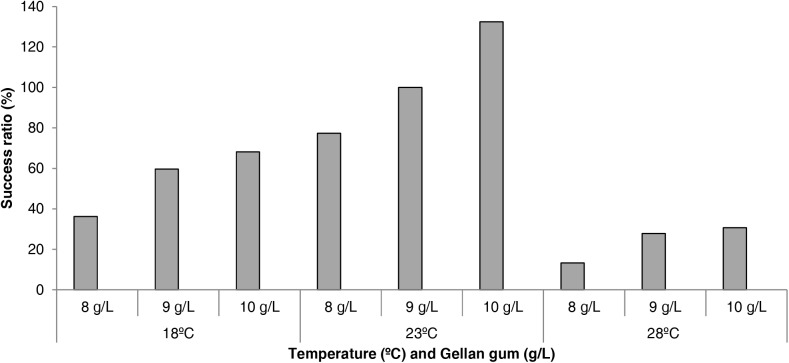
Success rate (%) in the process of plant conversion in *P. radiata* embryogenic cell lines maturated at three different temperatures (18, 23, and 28°C) and three gellan gum concentrations (8, 9, and 10 g L^-1^) with respect to standard maturation conditions (23°C and 9 gL^-1^ gellan gum, considered as 100% of success).

The effect of physical and chemical modification of culture media in the success of the conversion from EMs to plants is shown in Figure 7. An improvement in plant conversion was observed when the gellan gum concentration was increased at all temperatures tested. In this sense, 23°C and 10 gL^-1^ showed the best results improving by 132% the plant conversion process.

Somatic plants coming from different treatments during maturation stage did not show significant differences in *ex vitro* survival after 14 weeks growing in the greenhouse (Supplementary Material). However, highest rates were found in plants coming from EMs maturated at 28°C independently of the gellam gum concentration. Survival percentage increased parallel to the increment of temperature during the maturation stage.

### Endogenous Phytohormone Analyses

Detailed information on the contents of the 41 CKs and auxins in all samples analyzed is provided as Supplementary Material.

**FIGURE 8:**
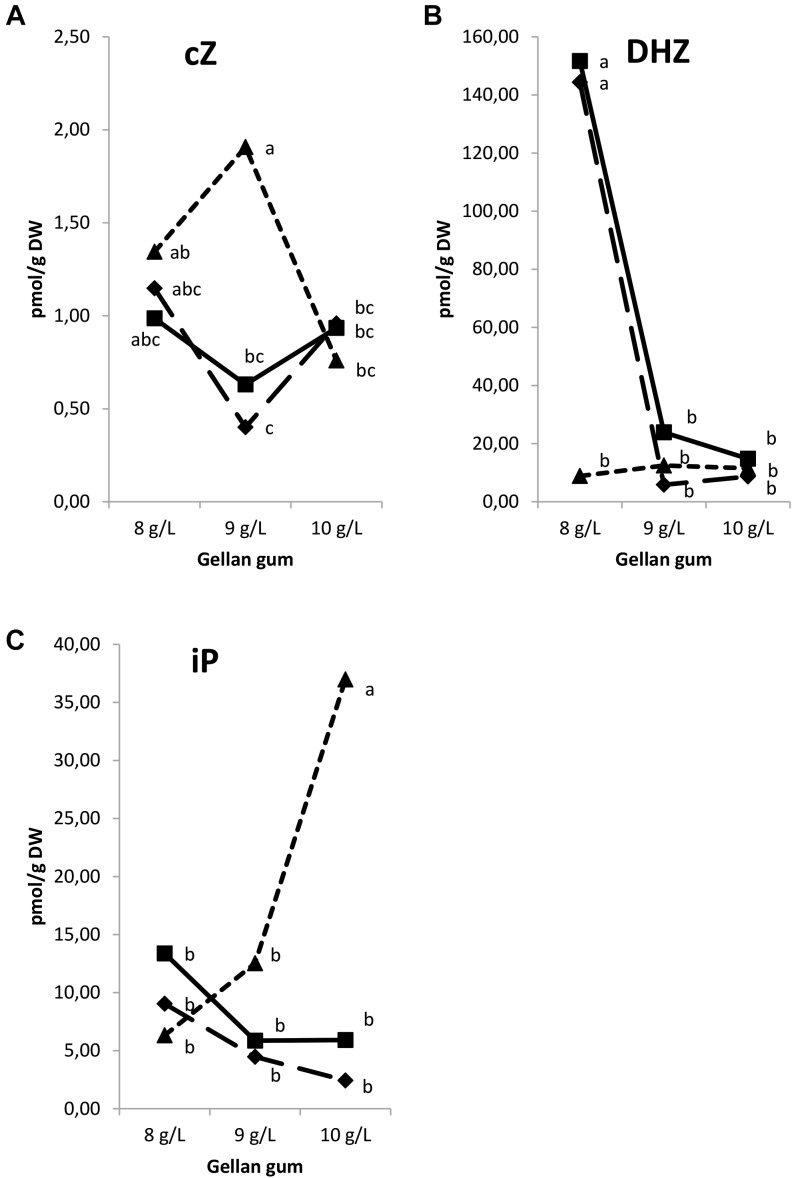
Endogenous levels (pmol g^-1^ DW) of the cytokinin bases in somatic embryos maturated at different temperatures in culture media solidified with different concentrations of gellan gum. *c*Z **(A)**, DHZ **(B)**, and iP **(C)** concentrations. Different letters indicate significant differences among treatments by Tukey’s *post hoc* test (α = 0.05). Triangle (28°C), square continuous line (23°C), and square discontinuous line (18°C).

Significant differences in CK bases were found among the levels of temperature, gellan gum (except for *c*Z) and the interaction between the tested variables. In all the Se analyzed the appearance of *t*Z was under the limit of detection. The concentration of *c*Z was the highest in Se maturated at the highest temperature with the standard concentration of gelrite (9 gL^-1^, Figure 8A). The levels of DHZ were significantly higher in somatic embryos cultured at 18 and 23°C with the lowest Gelrite concentration in the culture media (8 gL^-1^, Figure 8B). The iP concentration was the highest in Se developed in a culture media solidified with 10 gL^-1^ gelrite at 28°C (Figure 8C).

**FIGURE 9:**
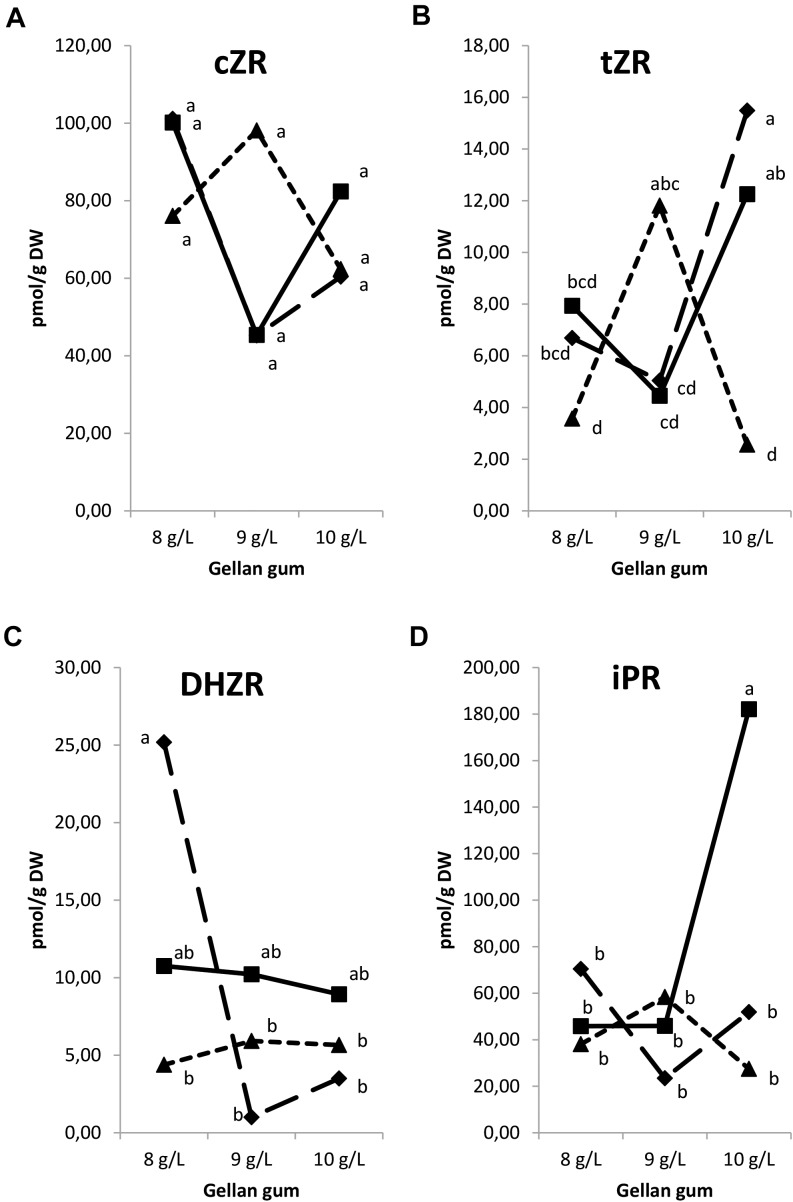
Endogenous levels (pmol g^1^ DW) of the cytokinin ribosides in somatic embryos maturated at different temperatures in culture media solidified with different concentrations of gellan gum. *cis*-zeatin riboside (cZR) **(A)**, *trans*-zeatin riboside (tZR) **(B)**, dihydrozeatin riboside (DHZR) **(C)**, and N6-isopentenyladenosine (iPR) **(D)** concentrations. Different letters indicate significant differences among treatments by Tukey’s *post hoc* test (α = 0.05). Triangle (28°C), square continuous line (23°C), and square discontinuous line (18°C).

Significant differences in CK ribosides (Figure 9) were found among the levels of gellan gum but no significant differences were observed among the levels of temperature for *t*ZR, *c*ZR, and DHZR; however, a significant interaction between temperature and agar was observed for all CK ribosides analyzed. *t*ZR levels increased significantly when the gelrite concentration increase from 9 to 10 in somatic embryos cultured at 18 and 23°C (Figure 9B). The highest DHZR concentration was found in Se cultured in presence of the lowest concentration of gelrite (8 gL^-1^) at 18°C (Figure 9C), the DHZR levels obtained this treatment did not present differences with those at standard temperature (23°C) regardless the gellan gum concentration. iPR levels were significantly higher in Se cultured at standard temperature in presence of the highest gelrite concentration (10 gL^-1^) (Figure 9D).

**FIGURE 10:**
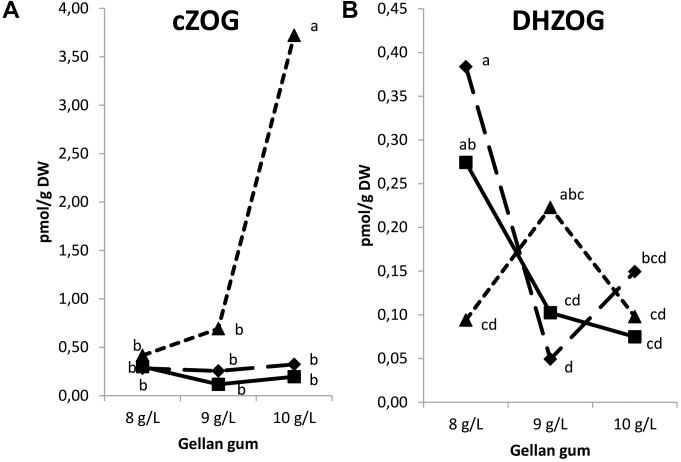
Endogenous levels (pmol g^-1^ DW) of the *O*-glucosides in somatic embryos maturated at different temperatures in culture media solidified with different concentrations of gellan gum. *cis*-zeatin O-glucoside (cZOG) **(A)**, dihydrozeatin *O*-glucoside (DHZOG) **(B)** concentrations. Different letters indicate significant differences among treatments by Tukey’s *post hoc* test (α = 0.05). Triangle (28°C), square continuous line (23°C), and square discontinuous line (18°C).

The levels of *t*ZRMP, *c*ZRMP, DHZRMP, and iPRMP were under detection limit in all the samples analyzed. Also, *t*ZOG, *t*ZROG, *c*ZROG, and DHZROG were under the limit of detection. Significant differences in CK *O*- glucosides were found among the levels of gellan gum and temperature (except for DHZOG), and for the interaction between the two variables. The CK *O*-glucosides were represented by a low amount of *c*ZOG and DHZOG. In this sense, Se maturated at the highest temperature and gelling agent concentration showed the highest levels of *c*ZOG (Figure 10A). On the contrary, DHZOG concentration was the highest in Se developed at the lowest temperature and gelrite concentration in the culture media (Figure 10B).

**FIGURE 11:**
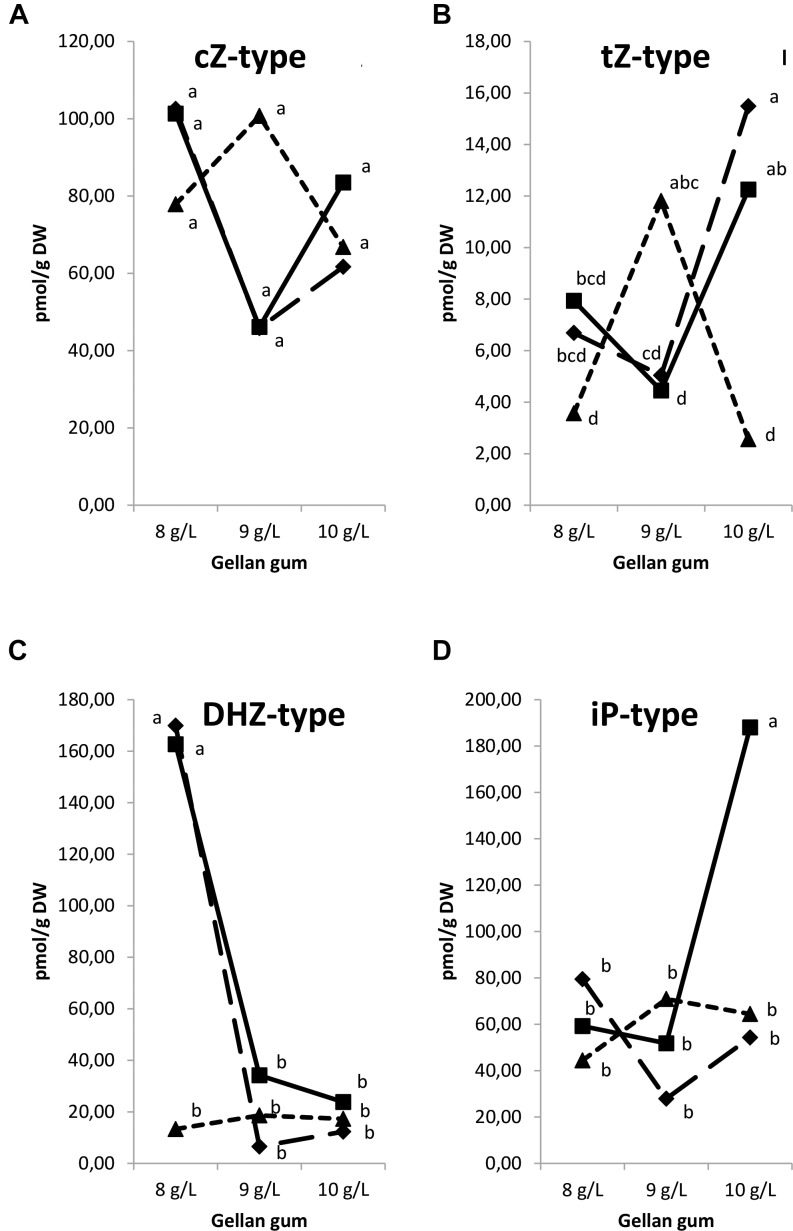
Endogenous levels (pmol g^-1^ DW) of different isoprenoid cytokinin types in somatic embryos maturated at different temperatures in culture media solidified with different concentrations of gellan gum. cZ-type **(A)**, tZ-type **(B)**, DHZ-type **(C)**, and iP-type **(D)** concentrations. Different letters indicate significant differences among treatments by Tukey’s *post hoc* test (α = 0.05). Triangle (28°C), square continuous line (23°C), and square discontinuous line (18°C).

Regarding the isoprenoid CK type, significant differences in CK types were found among the levels of temperature (except for *c*Z), gellan gum and the interaction between the tested variables. The *t*Z-type CK concentration was the highest in Se developed at 18 and 23°C with the highest concentration of gelling agent and at 28°C with the standard concentration of gelrite (Figure 11A). The lowest *c*Z-type CK concentration was observed in treatments at 18 and 23°C with the standard gelrite concentration (Figure 11B). The concentration of DHZ-type CKs was significantly higher in Se developed at 18 and 23°C in culture media solidified with 8 gL^-1^ of gelrite (Figure 11C). On the contrary, iP-types endogenous concentration was significantly higher in Se cultured with the highest concentration of gelling agent (10 gL^-1^) at standard temperature (23°C) than in Se from other treatments (Figure 11D).

**FIGURE 12:**
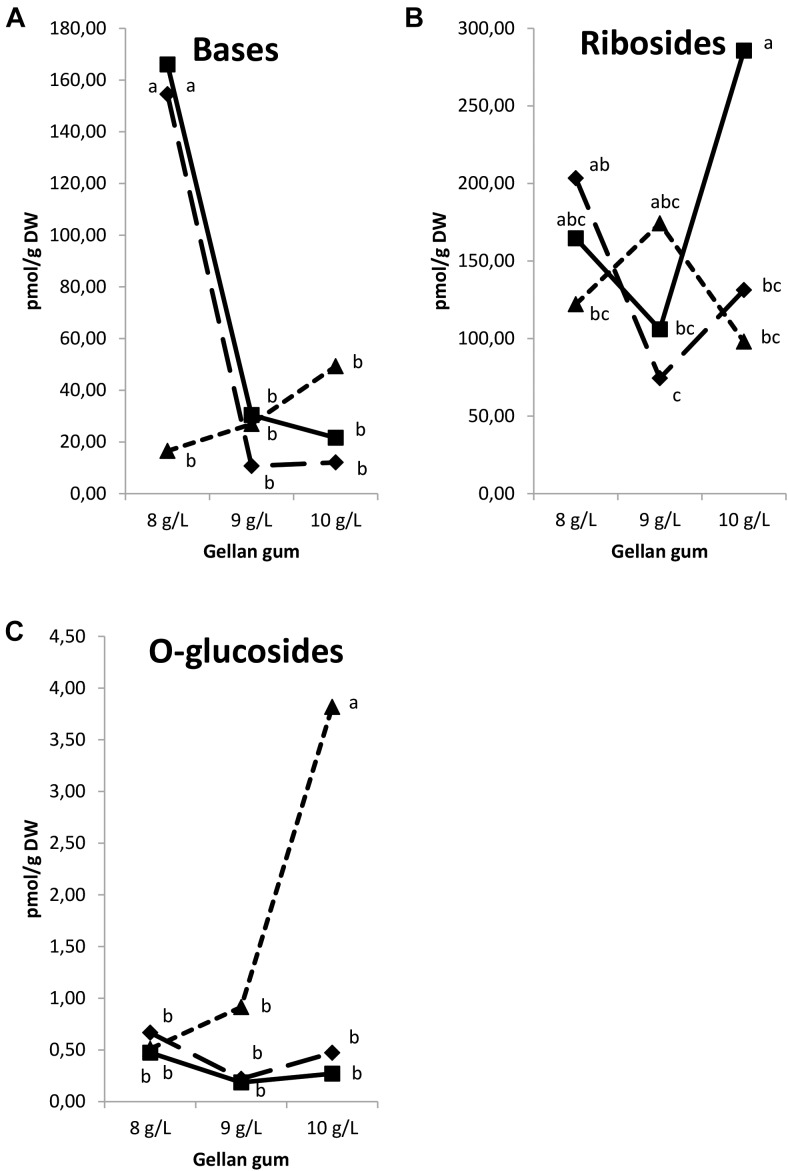
Endogenous levels (pmol g^-1^ DW) of the cytokinin groups in somatic embryos maturated at different temperatures in culture media solidified with different concentrations of gellan gum. Cytokinin Bases **(A)**, Ribosides **(B)**, and *O*-glucosides **(C)** concentrations. Different letters indicate significant differences among treatments by Tukey’s *post hoc* test (α = 0.05). Triangle (28°C), square continuous line (23°C), and square discontinuous line (18°C).

Analyzing the results by groups, CK nucleotides and *N*-glucosides were under the limit of detection. Significant differences in CK functional groups were found among the levels of temperature, gellan gum and the interaction between the tested variables. A significantly higher concentration of bases was found in Se developed at 18 and 23°C in culture media solidified with the lowest gelrite concentration (Figure 12A). Ribosides was the CK group with the most remarkable presence, the highest values for this group were obtained in Se cultured at standard temperature (23°C) and the highest gelrite concentration (Figure 12B). *O*-glucosides were significantly higher in Se cultured at the highest temperature and gelrite concentration (Figure 12C).

**FIGURE 13:**
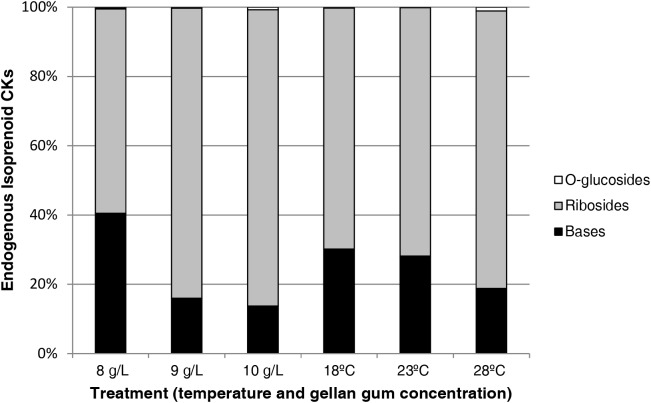
Relative abundances (expressed in percentage) of endogenous isoprenoid cytokinins in somatic embryos maturated at different temperatures in culture media solidified with different concentrations of gellan gum. Bases (black bar), Ribosides (gray bar), and *O*-glucosides (white bar).

Figure 13 shows the relative abundance of isoprenoid CKs Se by treatment. CK ribosides are the predominant group of cytokinins respect to bases and *O*-glucosides and a decrease in the bases content is observed parallel to an increase of gelrite concentration and temperature. Although the presence of aromatic type CKs (N^6^-benzyladenine, *meta*-topolin, *ortho*-topolin, and *para*-topolin) and their metabolites was also analyzed, theirs levels were under the limit of detection.

Taking a general look at the results presented, it was observed a similar trend for CK concentration in Se cultured at 18°C y 23°C when the agar concentration increased from 8 to 9 gL^-1^, except iPR, DHZR and *c*ZOG. When considering the results by CK-type or CK-metabolite this trend was also observed in all cases. At 28°C, an increase of bases and ribosides concentration was observed from 8 to 9 gL^-1^; in the case of iP, iPR, and *c*ZOG their concentration continued increasing significantly with gellan gum concentration (10 gL^-1^).

**FIGURE 14:**
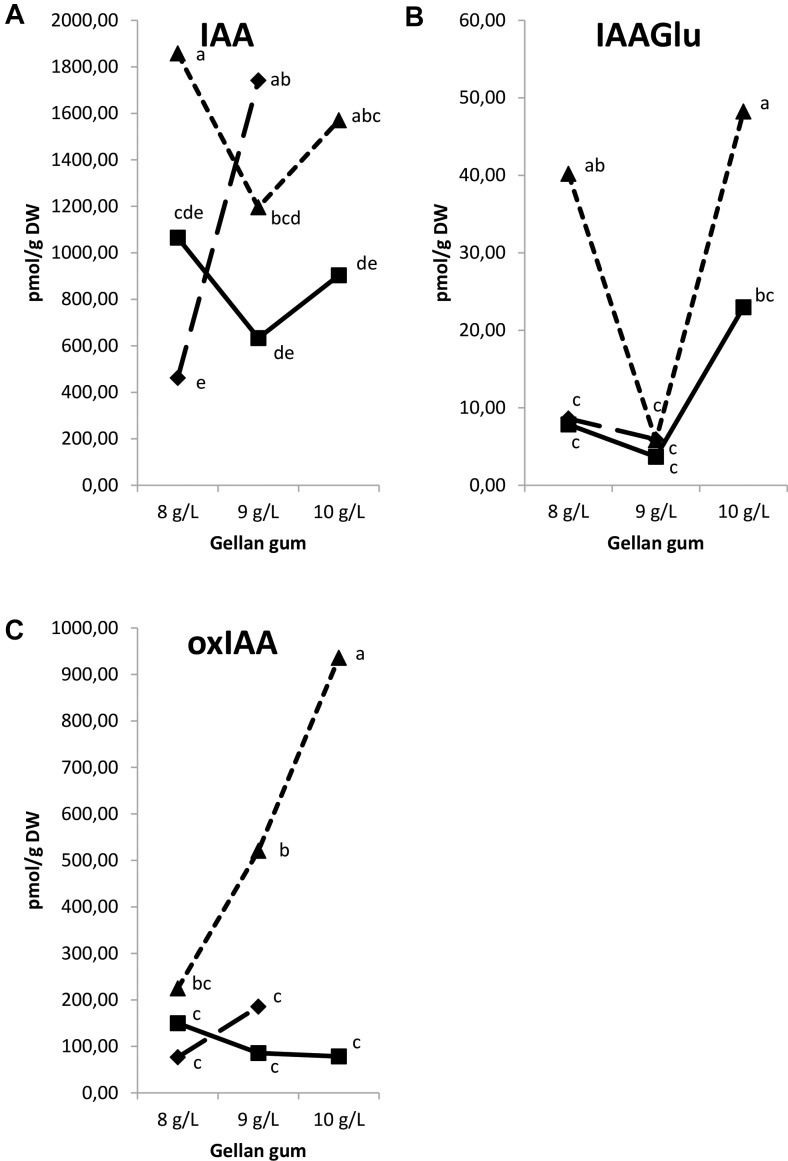
Endogenous levels (pmol g^-1^ DW) of indole-3-acetic acid (IAA) **(A)**, IAA-glutamate (IAA-Glu) **(B)**, 2-oxindole-3-acetic acid (oxIAA) **(C)** in somatic embryos IAA-glutamate (IAA-Glu), 2-oxindole-3-acetic acid (oxIAA) in somatic embryos maturated at different temperatures in culture media solidified with different concentrations of gellan gum. Different letters indicate significant differences among treatments by Tukey’s *post hoc* test (α = 0.05). Triangle (28°C), square continuous line (23°C), and square discontinuous line (18°C).

**FIGURE 15:**
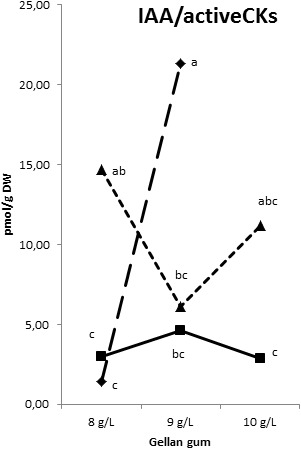
Indole-3-acetic acid/Cytokinin bases and ribosides ration in somatic embryos maturated at different temperatures in culture media solidified with different concentrations of gellan gum. Triangle (28°C), square continuous line (23°C), and square discontinuous line (18°C).

The IAA, 2-oxindole-3-acetic acid (oxIAA), and IAA-glutamate levels showed the highest levels in Se developed at the highest temperature with different concentrations of gelrite (Figures 14A–C). Moreover, Se cultured at 28°C in media with the highest gelrite concentration showed significantly higher levels of oxIAA and IAA-glutamate (Figures 14B,C). IAA-aspartate levels were under the limit of detection in all samples analyzed. When relation between IAA and active CKs (bases+ribosides) was evaluated, we could observe low values in Se developed at standard temperature (23°C) independently of the gelrite concentration in the culture media (Figure 15).

## Discussion

The inductive conditions which allow differentiated somatic cells to develop into competent dedifferentiated cells can be achieved by the addition of specific phytohormones, and/or by exposing the tissue to different stress factors (Leljak-Levanič et al., 2016). Moreover, water relations between the embryo and its environment play a regulatory role in embryo development, particularly during maturation (from Márquez-Martín et al., 2011) and for this reason, the water availability of maturation media was determined in our experiments. Radiata pine somatic embryos cultured in EDM medium supplemented with 10 gL^-1^ and stored at 28°C grew in an environment with the lowest water availability in comparison to the other conditions assayed. To this respect, in Douglas-fir, a reduction in water availability of the culture media provoked an increase in Se quality (Lelu-Walter et al., 2018) probably because it has been described that the reduction in water availability leads to obtain embryos with low water content similar to their zygotic counterparts (Morel et al., 2014).

The culture of embryogenic calli in media with an increased gelling agent concentration is a common procedure for the production of high quality mature embryos (Krajňáková et al., 2009; Troch et al., 2009; Buendía-González et al., 2012). Our results in *P. radiata* are in agreement with those obtained in *P. strobus* where the best maturation results were obtained increasing gellan gum concentration due to a shift in the developmental program of the culture, from proliferation of cells and early somatic embryos to the production of embryos at advanced stages (Klimaszewska et al., 2000).

Respect to the number of embryos developed, the temperature and gellan gum concentration affected significantly the number of Se per gram of EM. In this sense the temperature (28°C) and the agar concentration (10 gL^-1^) that provoked the lowest water availability led to the lowest and the highest number of Se, respectively. For this reason, it seems that water availability was not related to the success of the process at this stage. In this sense, there are two studies suggesting that under sub-optimal water availability, plants may take to a premature drought stress response and arrest or drastically reduce growth due to the effect on source–sink relationships through preferentially inhibiting nutrient acquisition processes more and earlier than necessary (Skirycz et al., 2011). It is clear that the water availability registered in our experiments is not sub-optimal for the maturation of EMs. In our laboratory, the effect of different physical and chemical conditions at the initiation stage of *P. radiata* and *P. halepensis* (García-Mendiguren et al., 2016; Pereira et al., 2016) as well as in proliferation stage of *P. halepensis* (Pereira et al., 2017) was studied and we found that the highest temperature seemed to produce a selective pressure as pointed by Fehér (2015); In this regard, Bonga et al. (2010) suggested that reducing or increasing temperatures may improve initiation and proliferation since temperature stress may promote cellular reprogramming. Our results are in agreement with de Almeida et al. (2014) where temperature had a significant influence on the direct SE capacity in coffee.

Regarding gellan gum concentration, some reports suggested that increasing the concentration of a gelling agent from 4 to 8 gL^-1^ (Teyssier et al., 2011) or from 4 to 9 gL^-1^ (Morel et al., 2014) promoted the maturation of the Se of some conifers. In this sense, Garin et al. (2000) demonstrated that the phytagel concentration was critical for the maturation of Se of five tested ECLs of *Pinus strobus*. The increase of phytagel concentration in the maturation media allowed obtaining well-developed Se capable of germination in various species of pines (Klimaszewska et al., 2009) and hybrid larch (Lelu-Walter and Pâques, 2009) probably due to the action in endogenous ABA modulation in mature somatic embryos (Morel et al., 2014). In summary, our results agree with abovementioned studies pointing that increasing gelling agent concentration is a good strategy to improve the Se yield in radiata pine.

The highest germination rates (above 90%) were obtained in Se coming from EMs maturated at 18 and 23°C, whereas maturation at 28°C led to lower germination rates, particularly when it was combined with 9 or 10 gL^-1^ gellan gum. Our results are not in agreement to Prewein et al. (2004) when they described that desiccation of the embryos due to decreased water availability in maturation media improves the germination frequency, probably by reducing the endogenous ABA level or by changing the sensitivity to ABA (Jiménez, 2005). Unlike observed by Morel et al. (2014) in maritime pine, we found no differences in germination rates with increasing concentration of agar, however, this could be due to differences in the range of concentrations used by these authors (4 and 9 gL^-1^ Phytagel) and us (8, 9, and 10 gL^-1^ Gelrite).

An improvement in plant conversion when the gellan gum concentration was increased at all temperatures tested was observed. However, 23°C showed the best results improving by 132% the plant conversion process. Summarizing, increasing the gellan gum concentration in the maturation stage improved the SE process in all radiata pine cultures independently of the temperature of storage obtaining the best results when somatic embryos were submitted to maturation process at control temperature (23°C). So, higher temperature and gellan gum concentration applied at maturation stage did not result in an increment of the efficiency of SE process like it was observed when this stressful condition was applied at initiation stage (García-Mendiguren et al., 2016). Moreover, *ex vitro* survival did not show significant differences although the highest percentages were obtained in somatic plants coming from EMs maturated at 28°C.

There are only a few reports describing how the contents of different endogenous hormones are altered by different environmental conditions. The relative abundance of different CKs can vary greatly between plant species, tissues and developmental stages, and depends on the environmental conditions (Frébort et al., 2011). In our experiments, the prevailing isoforms of CKs were *cis*-isomers of Z and ZR according with Zur et al. (2015) in microspore embryogenesis in triticale. Based on studies with *Arabidopsis*, *cis*-isomers were regarded as CK derivatives without any or with low biological activity. Further studies with non-model plants, like pines, showed that *cis*-isomers can be the dominant form of CK in specific plant organs and/or stages of development (Emery et al., 1998: Vyroubalová et al., 2009; Kudo et al., 2012). To this respect, Gajdošová et al. (2011) proposed that *c*Z can be qualified as a regulator of CK responses in plants under growth-limiting conditions. Our results only showed significantly differences in *c*Z in Se cultured with 9 gL^-1^ of gelrite at the highest temperature (28°C) with the others analyzed.

Highest amount of bases, the active forms of cytokinins (Klemš et al., 2011), were found in Se developed under 18 and 23°C and low gellan gum concentration, treatments showing the highest water availability. So, our results are in agreement to other authors describing that a decrease in water availability could be related to the rapidly utilization of CK bases and tightly regulated by mechanisms that prevent their accumulation at high levels (Moncaleán et al., 2005; Stirk et al., 2005).

CK ribosides, mainly represented by *c*ZR and iPR, were higher in the treatment that led to the highest somatic embryo production and success rate (23°C and 10 gL^-1^). In terms of metabolic groups, ribosides were the predominant group of cytokinins and its percentage respect to bases and *O*-glucosides increased in parallel with the decrease of water availability. Nevertheless, in peach seeds, phosphates and ribosides of the DHZ and iP-type are the predominant CKs and functions like embryo formation and growth, division of endosperm primary nucleus, endosperm cellularization and endosperm cell division have been attributed to them (Arnau et al., 1999). So, opposite to Pacheco de Freitas et al. (2016) active CK levels seem to be important during the later stages of embryo development and maturation showing large amounts of these phytohormones in treatments with high production of mature embryos.

CK *O*-glucosides (reversible forms of storage), particularly *c*ZOG, were highest in those embryos coming from EMs maturated in the most stressful conditions (28°C and 10 gL^-1^ gelrite, lowest water availability). However, CK *O*-glucosides were found in minute amounts (below 4 pmolg^-1^ DW), for instance Montalbán et al. (2011) reported *O*-glucoside concentrations above 240 pmolg^-1^ DW in *P. radiata* vegetative buds.

Low content of IAA and iP were obtained in Se treated with the highest gelrite concentration at the standard temperature (23°C). In this sense, in cotton a high content of both groups of phytohormones were related to the redifferentiation stage that leads to embryogenesis induction (Zeng et al., 2007). Somleva et al. (1995) and Sáenz et al. (2003) have found an inverse relationship between cytokinins and the embryogenic response; in this sense, the treatment that provoked the highest success in the SE process (23°C, 10 gL^-1^ gelrite) showed a low level of IAA/bases+ribosides CKs relation. So it seems that IAA can play an important role in radiata pine SE maturation process; this conclusion is in agreement to the postulation that auxin metabolism (biosynthesis, conjugation, degradation), intercellular transport, and signaling is crucial in coordinating the morphogenesis and development of plant reproductive organs, including the formation of embryos (Benková et al., 2003; Bernardi et al., 2012). The accumulation of endogenous IAA in response to abiotic stress may have a mediating role during SE (Pěnčík et al., 2015; Nic-Can et al., 2016).

It seems that the differences found in specific CKs analyzed did not provoke changes in maturation rates. Our results suggest that none of phytohormones found acts alone in the acquisition of embryogenic maturation capacity; it seems that the dynamic and complementary actions of the auxin and cytokinin pathways regulate several developmental processes and their ability to crosstalk makes them ideal candidates for mediating stress-adaptation responses according with Bielach et al. (2017) as well as initiating various signal transduction pathways (Kohli et al., 2013). In this regard, comparing the endogenous levels of IAA, Z, ZR, iP, and iPR in several embryogenic habituated callus lines of *Asparagus officinalis*, no correlation between embryogenic potential and endogenous hormone levels could be found (Limanton-Grevet et al., 2000). These and other findings demonstrate that there is no direct correlation in endogenous hormone content and embryogenic competence of different genotypes, or between competent and non-competent genotypes, which suggests the involvement of additional, hormone-related internal mechanisms for embryogenic competence regulation and regeneration ability *in vitro* (Leljak-Levanič et al., 2016). However, from our results, it seems clear that low levels in the relation between IAA and CK bases+ribosides are related to highest success of SE process in radiata pine.

Further studies increasing the temperatures during shorter periods of time can help us to go further in the understanding of the mechanisms involved in SE in *Pinus radiata* as well as the role of phytohormones in this process.

## Author Contributions

PM and IM conceived and planned the experiments. PM, OG-M, and IM carried out maturation experiments. OG-M, ON, and MS carried out phytohormone analyses. IM, MU, and TG carried out the statistical analyses. All authors provided critical feedback and helped to shape the research, analyses, and manuscript.

## Conflict of Interest Statement

The authors declare that the research was conducted in the absence of any commercial or financial relationships that could be construed as a potential conflict of interest.
