# Supplementary material for: Temperature and Water Availability During Maturation Affect the Cytokinins and Auxins Profile of Radiata Pine Somatic Embryos
Source: Front Plant Sci. 2018 Dec 20;9:1898. doi: 10.3389/fpls.2018.01898 (PMC6306442; doi:10.3389/fpls.2018.01898)
Supplement: Supplementary file 1 [file Table_1.docx]

Supplementary Material

**Temperature and water availability during maturation affect the cytokinins and auxins profile of radiata pine somatic embryos**

Moncaleán P., García-Mendiguren O., Novák O., Strand M., Goicoa T., Ugarte M.D., Montalbán I.A.*

*** Correspondence:** Itziar A. Montalbán: imontalban@neiker.eus

# Supplementary Tables

**Table 1.** Analysis of variance of the water availability (mg) in nine maturation media for *Pinus radiata*, containing different gellan gum concentrations (8, 9 and 10 gL^-1^) and stored at different temperatures (18, 23 and 28ºC).

| Source | d*f* | F value | *p* value |
| --- | --- | --- | --- |
| Temperature (T) | 2 | 34.781 | <0.001 |
| Gellan gum (G) | 2 | 9.298 | <0.001 |
| T x G | 4 | 0.770 | 0.554 |

**Table 2.** Analysis of deviance of the logistic regression for the number of *Pinus radiata* somatic embryos per gram of embryonal mass in nine maturation media, containing different gellan gum concentrations (8, 9 and 10 gL^-1^) and stored at different temperatures (18, 23 and 28ºC).

| Source | d*f* | χ^2^ test | *p* value |
| --- | --- | --- | --- |
| Temperature (T) | 2 | 121.633 | <0.001 |
| Gellan gum (G) | 2 | 70.068 | <0.001 |
| T x G | 4 | 5.039 | 0.283 |

**Table 3.** Analysis of deviance of the logistic regression for germination rates of *Pinus radiata* somatic embryos from nine maturation media, containing different gellan gum concentrations (8, 9 and 10 gL^-1^) and stored at different temperatures (18, 23 and 28ºC).

| Source | d*f* | χ^2^ test | *p* value |
| --- | --- | --- | --- |
| Temperature (T) | 2 | 108.649 | <0.001 |
| Gellan gum (G) | 2 | 7.939 | <0.05 |
| T x G | 4 | 14.560 | <0.01 |

**Table 4.** Analysis of variance of endogenous cytokinin bases [*cis*-zeatin (cZ), dihydrozeatin (DHZ), N6-isopentenyladenine (iP) (pmol g^−1^ DW)] in *Pinus radiata* somatic embryos from nine different maturation treatments (maturated at 18, 23 and 28ºC and at 8, 9 and 10gL^-1^ gellan gum).

| Source | Variable | d*f* | F value | *p* value |
| --- | --- | --- | --- | --- |
| Temperature (T) | cZ | 2 | 7.922 | <0.01 |
|  | DHZ | 2 | 16.907 | <0.001 |
|  | iP | 2 | 19.509 | <0.001 |
|  |  |  |  |  |
| Gellan gum (G) | cZ | 2 | 1.902 | 0.178 |
|  | DHZ | 2 | 57.568 | <0.001 |
|  | iP | 2 | 6.064 | <0.05 |
|  |  |  |  |  |
| T x G | cZ | 4 | 7.438 | <0.01 |
|  | DHZ | 4 | 15.509 | <0.001 |
|  | iP | 4 | 16.612 | <0.001 |

**Table 5.** Analysis of variance of endogenous cytokinin ribosides [*cis*-zeatin riboside (cZR), dihydrozeatin riboside (DHZR), N6-isopentenyladenosine (iPR) and *trans*-zeatin riboside (tZR) (pmol g^−1^ DW)] in *Pinus radiata* somatic embryos from nine different maturation treatments (maturated at 18, 23 and 28ºC and at 8, 9 and 10gL^-1^ gellan gum).

| Source | Variable | d*f* | F value | *p* value |
| --- | --- | --- | --- | --- |
| Temperature (T) | tZR | 2 | 3.143 | 0.069 |
|  | cZR | 2 | 0.623 | 0.548 |
|  | DHZR | 2 | 1.547 | 0.241 |
|  | iPR | 2 | 18.946 | <0.001 |
|  |  |  |  |  |
| Gellan gum (G) | tZR | 2 | 6.280 | <0.01 |
|  | cZR | 2 | 4.567 | <0.05 |
|  | DHZR | 2 | 4.154 | <0.05 |
|  | iPR | 2 | 13.976 | <0.001 |
|  |  |  |  |  |
| T x G | tZR | 4 | 13.698 | <0.001 |
|  | cZR | 4 | 3.632 | <0.05 |
|  | DHZR | 4 | 4.219 | <0.05 |
|  | iPR | 4 | 23.416 | <0.001 |

**Table 6.** Analysis of variance of endogenous cytokinin *O*-glucosides [cis-zeatin O-glucoside (cZOG), dihydrozeatin *O*-glucoside (DHZOG) (pmol g^−1^ DW)] in *Pinus radiata* somatic embryos from nine different maturation treatments (maturated at 18, 23 and 28ºC and at 8, 9 and 10gL^-1^ gellan gum).

| Source | Variable | d*f* | F value | *p* value |
| --- | --- | --- | --- | --- |
| Temperature (T) | cZOG | 2 | 22.143 | <0.001 |
|  | DHZOG | 2 | 2.471 | 0.116 |
|  |  |  |  |  |
| Gellan gum (G) | cZOG | 2 | 13.583 | <0.001 |
|  | DHZOG | 2 | 14.490 | <0.001 |
|  |  |  |  |  |
| T x G | cZOG | 4 | 13.584 | <0.001 |
|  | DHZOG | 4 | 10.846 | <0.001 |

**Table 7.** Analysis of variance of endogenous isoprenoid cytokinin pools [cis-zeatin pool (cZ-type), dihydrozeatin pool (DHZ-type), N6-isopentenyladenine pool (iP-type) and trans-zeatin pool (tZ-type) (pmol g^−1^ DW)] in *Pinus radiata* somatic embryos from nine different maturation treatments (maturated at 18, 23 and 28ºC and at 8, 9 and 10gL^-1^ gellan gum).

| Source | Variable | d*f* | F value | *p* value |
| --- | --- | --- | --- | --- |
| Temperature (T) | tZ-type | 2 | 3.747 | <0.05 |
|  | cZ-type | 2 | 0.612 | 0.553 |
|  | DHZ-type | 2 | 15.042 | <0.001 |
|  | iP-type | 2 | 14.363 | <0.001 |
|  |  |  |  |  |
| Gellan gum (G) | tZ-type | 2 | 6.459 | <0.01 |
|  | cZ-type | 2 | 4.259 | <0.05 |
|  | DHZ-type | 2 | 50.588 | <0.001 |
|  | iP-type | 2 | 17.502 | <0.001 |
|  |  |  |  |  |
| T x G | tZ-type | 4 | 14.549 | <0.001 |
|  | cZ-type | 4 | 3.546 | <0.05 |
|  | DHZ-type | 4 | 14.299 | <0.001 |
|  | iP-type | 4 | 17.243 | <0.001 |

**Table 7.** Analysis of variance of endogenous cytokinin pools based on structural and functional forms [bases, ribosides and *O*-glucosides (pmol g^−1^ DW)] in *Pinus radiata* somatic embryos from nine different maturation treatments (maturated at 18, 23 and 28ºC and at 8, 9 and 10gL^-1^ gellan gum).

| Source | Variable | d*f* | F value | *p* value |
| --- | --- | --- | --- | --- |
| Temperature (T) | Bases | 2 | 9.450 | <0.01 |
|  | Ribosides | 2 | 4.206 | <0.05 |
|  | O-glucosides | 2 | 23.805 | <0.001 |
|  |  |  |  |  |
| Gellan gum (G) | Bases | 2 | 52.845 | <0.001 |
|  | Ribosides | 2 | 3.908 | <0.05 |
|  | O-glucosides | 2 | 13.428 | <0.001 |
|  |  |  |  |  |
| T x G | Bases | 4 | 20.951 | <0.001 |
|  | Ribosides | 4 | 9.132 | <0.001 |
|  | O-glucosides | 4 | 14.362 | <0.001 |

**Table 8.** Endogenous levels (pmol g^−1^ DW) of cytokinin bases in somatic embryos from nine different treatments (maturated at 18, 23 and 28ºC and at 8, 9 and 10gL^-1^ Gelrite®). Mean ± standard error; BA, N6-benzyladenine; cZ, *cis*-zeatin; DHZ, dihydrozeatin; iP, N6-isopentenyladenine; mT, *meta*-topolin; oT, ortho-topolin; pT, *para*-topolin; tZ, *trans*-zeatin.

| **Treatment** | **tZ** |  | **cZ** | | |  | **DHZ** | | | **iP** | | |  |  | **BA** |  | **oT** |  | **mT** |  | **pT** |
| --- | --- | --- | --- | --- | --- | --- | --- | --- | --- | --- | --- | --- | --- | --- | --- | --- | --- | --- | --- | --- | --- |
| 18ºC 8 g/L | <LOD |  | 1.15 | ± | 0.21 |  | 144.34 | ± | 20.25 | 9.04 | ± | 1.47 |  |  | <LOD |  | <LOD |  | <LOD |  | <LOD |
| 18ºC 9 g/L | <LOD |  | 0.40 | ± | 0.05 |  | 5.83 | ± | 1.509 | 4.47 | ± | 0.52 |  |  | <LOD |  | <LOD |  | <LOD |  | <LOD |
| 18ºC 10 g/L | <LOD |  | 0.96 | ± | 0.05 |  | 8.68 | ± | 0.89 | 2.43 | ± | 0.34 |  |  | <LOD |  | <LOD |  | <LOD |  | <LOD |
| 23ºC 8 g/L | <LOD |  | 0.99 | ± | 0.12 |  | 151.67 | ± | 27.91 | 13.38 | ± | 2.65 |  |  | <LOD |  | <LOD |  | <LOD |  | <LOD |
| 23ºC 9 g/L | <LOD |  | 0.63 | ± | 0.09 |  | 23.90 | ± | 4.79 | 5.87 | ± | 0.80 |  |  | <LOD |  | <LOD |  | <LOD |  | <LOD |
| 23ºC 10 g/L | <LOD |  | 0.93 | ± | 0.16 |  | 14.80 | ± | 2.50 | 5.92 | ± | 1.28 |  |  | <LOD |  | <LOD |  | <LOD |  | <LOD |
| 28ºC 8 g/L | <LOD |  | 1.34 | ± | 0.14 |  | 8.85 | ± | 0.87 | 6.32 | ± | 0.66 |  |  | <LOD |  | <LOD |  | <LOD |  | <LOD |
| 28ºC 9 g/L | <LOD |  | 1.91 | ± | 0.36 |  | 12.49 | ± | 2.33 | 12.53 | ± | 2.28 |  |  | <LOD |  | <LOD |  | <LOD |  | <LOD |
| 28ºC 10 g/L | <LOD |  | 0.76 | ± | 0.16 |  | 11.54 | ± | 2.32 | 36.97 | ± | 7.04 |  |  | <LOD |  | <LOD |  | <LOD |  | <LOD |

**Table 9.** Endogenous levels (pmol g^−1^ DW) of cytokinin ribosides in somatic embryos from nine different treatments (maturated at 18, 23 and 28ºC and at 8, 9 and 10gL^-1^ Gelrite®). Mean ± standard error; BAR, N6-benzyladenosine; cZR, *cis*-zeatin riboside; DHZR, dihydrozeatin riboside; iPR, N6-isopentenyladenosine; mTR, *meta*-topolin riboside; oTR, *ortho*-topolin riboside; pTR, *para*-topolin riboside; tZR, *trans*-zeatin riboside.

| **Treatment** | **tZR** | | |  | **cZR** | | |  | **DHZR** | | |  | **iPR** | | |  | **BAR** |  | **oTR** |  | **mTR** |  | **pTR** |
| --- | --- | --- | --- | --- | --- | --- | --- | --- | --- | --- | --- | --- | --- | --- | --- | --- | --- | --- | --- | --- | --- | --- | --- |
| 18ºC 8 g/L | 6.69 | ± | 0.45 |  | 101.11 | ± | 18.86 |  | 25.18 | ± | 9.98 |  | 70.41 | ± | 11.09 |  | <LOD |  | <LOD |  | <LOD |  | <LOD |
| 18ºC 9 g/L | 5.04 | ± | 0.49 |  | 45.26 | ± | 7.52 |  | 0.67 | ± | 0.34 |  | 23.43 | ± | 2.88 |  | <LOD |  | <LOD |  | <LOD |  | <LOD |
| 18ºC 10 g/L | 15.49 | ± | 2.42 |  | 60.41 | ± | 6.24 |  | 3.50 | ± | 0.87 |  | 51.87 | ± | 7.82 |  | <LOD |  | <LOD |  | <LOD |  | <LOD |
| 23ºC 8 g/L | 7.93 | ± | 1.34 |  | 100.13 | ± | 13.68 |  | 10.75 | ± | 0.92 |  | 45.82 | ± | 6.43 |  | <LOD |  | <LOD |  | <LOD |  | <LOD |
| 23ºC 9 g/L | 4.45 | ± | 0.94 |  | 45.36 | ± | 7.32 |  | 10.22 | ± | 0.85 |  | 45.94 | ± | 7.80 |  | <LOD |  | <LOD |  | <LOD |  | <LOD |
| 23ºC 10 g/L | 12.25 | ± | 1.85 |  | 82.37 | ± | 15.03 |  | 8.93 | ± | 2.36 |  | 182.11 | ± | 23.86 |  | <LOD |  | <LOD |  | <LOD |  | <LOD |
| 28ºC 8 g/L | 3.57 | ± | 0.69 |  | 76.08 | ± | 15.25 |  | 4.38 | ± | 1.89 |  | 38.09 | ± | 7.52 |  | <LOD |  | <LOD |  | <LOD |  | <LOD |
| 28ºC 9 g/L | 11.81 | ± | 2.29 |  | 98.11 | ± | 14.54 |  | 5.92 | ± | 0.59 |  | 58.38 | ± | 4.76 |  | <LOD |  | <LOD |  | <LOD |  | <LOD |
| 28ºC 10 g/L | 2.56 | ± | 0.49 |  | 62.34 | ± | 11.25 |  | 5.66 | ± | 1.61 |  | 27.46 | ± | 4.54 |  | <LOD |  | <LOD |  | <LOD |  | <LOD |

**Table 10.** Endogenous levels (pmol g^−1^ DW) of the cytokinin nucleotides in somatic embryos from nine different treatments (maturated at 18, 23 and 28ºC and at 8, 9 and 10gL^-1^ Gelrite®). BARMP, N6-benzyladenosine-5´-monophosphate; cZRMP, *cis*-zeatin riboside-5´-monophosphate; DHZRMP, dihydrozeatin riboside-5´-monophosphate; iPRMP, N6-isopentenyladenosine-5´-monophosphate; tZRMP, *trans*-zeatin riboside-5´-monophosphate.

| **Treatment** |  |  | **tZRMP** |  |  | **cZRMP** |  |  | **DHZRMP** |  |  | **iPRMP** |  | **BARMP** |
| --- | --- | --- | --- | --- | --- | --- | --- | --- | --- | --- | --- | --- | --- | --- |
| 18ºC 8 g/L |  |  | <LOD |  |  | <LOD |  |  | <LOD |  |  | <LOD |  | <LOD |
| 18ºC 9 g/L |  |  | <LOD |  |  | <LOD |  |  | <LOD |  |  | <LOD |  | <LOD |
| 18ºC 10 g/L |  |  | <LOD |  |  | <LOD |  |  | <LOD |  |  | <LOD |  | <LOD |
| 23ºC 8 g/L |  |  | <LOD |  |  | <LOD |  |  | <LOD |  |  | <LOD |  | <LOD |
| 23ºC 9 g/L |  |  | <LOD |  |  | <LOD |  |  | <LOD |  |  | <LOD |  | <LOD |
| 23ºC 10 g/L |  |  | <LOD |  |  | <LOD |  |  | <LOD |  |  | <LOD |  | <LOD |
| 28ºC 8 g/L |  |  | <LOD |  |  | <LOD |  |  | <LOD |  |  | <LOD |  | <LOD |
| 28ºC 9 g/L |  |  | <LOD |  |  | <LOD |  |  | <LOD |  |  | <LOD |  | <LOD |
| 28ºC 10 g/L |  |  | <LOD |  |  | <LOD |  |  | <LOD |  |  | <LOD |  | <LOD |

**Table 11.** Endogenous levels (pmol g^−1^ DW) of cytokinin *O*-glucosides in somatic embryos from nine different treatments (maturated at 18, 23 and 28ºC and at 8, 9 and 10gL^-1^ Gelrite®). Mean ± standard error; cZOG, *cis*-zeatin *O*-glucoside; cZROG, cis-zeatin riboside *O*-glucoside; DHZOG, dihydrozeatin *O*-glucoside; DHZROG, dihydrozeatin riboside *O*-glucoside; tZOG, *trans*-zeatin *O*-glucoside; tZROG, *trans*-zeatin riboside *O*-glucoside.

| **Treatment** |  | **tZOG** |  | **tZROG** |  | **cZOG** | | |  | **cZROG** |  | **DHZOG** | | |  | **DHZROG** |  |
| --- | --- | --- | --- | --- | --- | --- | --- | --- | --- | --- | --- | --- | --- | --- | --- | --- | --- |
| 18ºC 8 g/L |  | <LOD |  | <LOD |  | 0.28 | ± | 0.04 |  | <LOD |  | 0.38 | ± | 0.07 |  | <LOD |  |
| 18ºC 9 g/L |  | <LOD |  | <LOD |  | 0.17 | ± | 0.09 |  | <LOD |  | 0.05 | ± | 0.01 |  | <LOD |  |
| 18ºC 10 g/L |  | <LOD |  | <LOD |  | 0.32 | ± | 0.06 |  | <LOD |  | 0.15 | ± | 0.02 |  | <LOD |  |
| 23ºC 8 g/L |  | <LOD |  | <LOD |  | 0.20 | ± | 0.10 |  | <LOD |  | 0.27 | ± | 0.01 |  | <LOD |  |
| 23ºC 9 g/L |  | <LOD |  | <LOD |  | 0.12 | ± | 0.01 |  | <LOD |  | 0.07 | ± | 0.04 |  | <LOD |  |
| 23ºC 10 g/L |  | <LOD |  | <LOD |  | 0.20 | ± | 0.04 |  | <LOD |  | 0.07 | ± | 0.01 |  | <LOD |  |
| 28ºC 8 g/L |  | <LOD |  | <LOD |  | 0.42 | ± | 0.10 |  | <LOD |  | 0.09 | ± | 0.02 |  | <LOD |  |
| 28ºC 9 g/L |  | <LOD |  | <LOD |  | 0.69 | ± | 0.03 |  | <LOD |  | 0.22 | ± | 0.03 |  | <LOD |  |
| 28ºC 10 g/L |  | <LOD |  | <LOD |  | 3.72 | ± | 0.79 |  | <LOD |  | 0.10 | ± | 0.02 |  | <LOD |  |

**Table 12.** Endogenous levels (pmol g^−1^ DW) of CK N-glucosides in somatic embryos from nine different treatments (maturated at 18, 23 and 28ºC and at 8, 9 and 10gL-1 Gelrite®). BA9G, N6-benzyladenine-9-glucoside; cZ9G, *cis*-zeatin-9-glucoside; DHZ7G, dihydrozeatin-7-glucoside; DHZ9G, dihydrozeatin-9-glucoside; iP7G, N6-isopentenyladenine-7-glucoside; iP9G, N6-isopentenyladenine-9-glucoside; mT9G, *meta*-topolin-9-glucoside; oT9G, *ortho*-topolin-9-glucoside; tZ7G, *trans*-zeatin-7-glucoside; tZ9G, *trans*-zeatin-9-glucoside.

| **Treatment** |  | **tZ7G** |  | **tZ9G** |  | **cZ9G** |  | **DHZ7G** |  | **DHZ9G** |  | **iP7G** |  | **iP9G** |  | **BA9G** |  | **oT9G** |  | **mT9G** |
| --- | --- | --- | --- | --- | --- | --- | --- | --- | --- | --- | --- | --- | --- | --- | --- | --- | --- | --- | --- | --- |
| 18ºC 8 g/L |  | <LOD |  | <LOD |  | <LOD |  | <LOD |  | <LOD |  | <LOD |  | <LOD |  | <LOD |  | <LOD |  | <LOD |
| 18ºC 9 g/L |  | <LOD |  | <LOD |  | <LOD |  | <LOD |  | <LOD |  | <LOD |  | <LOD |  | <LOD |  | <LOD |  | <LOD |
| 18ºC 10 g/L |  | <LOD |  | <LOD |  | <LOD |  | <LOD |  | <LOD |  | <LOD |  | <LOD |  | <LOD |  | <LOD |  | <LOD |
| 23ºC 8 g/L |  | <LOD |  | <LOD |  | <LOD |  | <LOD |  | <LOD |  | <LOD |  | <LOD |  | <LOD |  | <LOD |  | <LOD |
| 23ºC 9 g/L |  | <LOD |  | <LOD |  | <LOD |  | <LOD |  | <LOD |  | <LOD |  | <LOD |  | <LOD |  | <LOD |  | <LOD |
| 23ºC 10 g/L |  | <LOD |  | <LOD |  | <LOD |  | <LOD |  | <LOD |  | <LOD |  | <LOD |  | <LOD |  | <LOD |  | <LOD |
| 28ºC 8 g/L |  | <LOD |  | <LOD |  | <LOD |  | <LOD |  | <LOD |  | <LOD |  | <LOD |  | <LOD |  | <LOD |  | <LOD |
| 28ºC 9 g/L |  | <LOD |  | <LOD |  | <LOD |  | <LOD |  | <LOD |  | <LOD |  | <LOD |  | <LOD |  | <LOD |  | <LOD |
| 28ºC 10 g/L |  | <LOD |  | <LOD |  | <LOD |  | <LOD |  | <LOD |  | <LOD |  | <LOD |  | <LOD |  | <LOD |  | <LOD |

**Table 13.** Endogenous isoprenoid cytokinin pools (pmol g^-1^ DW) in somatic embryos from nine different treatments (maturated at 18, 23 and 28ªC and at 8, 9 and 10gL^-1^ Gelrite®) mean ± standard error. Endogenous aromatic cytokinins were under the limit of detection.

| **Treatment** |  | **tZ-type** | | |  | **cZ-type** | | |  | **DHZ-type** | | |  | **iP-type** | | |  |
| --- | --- | --- | --- | --- | --- | --- | --- | --- | --- | --- | --- | --- | --- | --- | --- | --- | --- |
| 18ºC 8 g/L |  | 6.69 | ± | 0.45 |  | 102.54 | ± | 19.06 |  | 169.90 | ± | 27.46 |  | 79.44 | ± | 9.64 |  |
| 18ºC 9 g/L |  | 5.04 | ± | 0.49 |  | 45.83 | ± | 7.66 |  | 6.55 | ± | 0.77 |  | 27.90 | ± | 3.23 |  |
| 18ºC 10 g/L |  | 15.49 | ± | 2.42 |  | 61.69 | ± | 6.31 |  | 12.33 | ± | 0.98 |  | 54.30 | ± | 7.98 |  |
| 23ºC 8 g/L |  | 7.93 | ± | 1.34 |  | 101.32 | ± | 13.85 |  | 162.69 | ± | 28.77 |  | 59.19 | ± | 7.79 |  |
| 23ºC 9 g/L |  | 4.45 | ± | 0.94 |  | 46.11 | ± | 7.41 |  | 34.19 | ± | 5.48 |  | 51.81 | ± | 8.15 |  |
| 23ºC 10 g/L |  | 12.25 | ± | 1.85 |  | 83.50 | ± | 15.21 |  | 23.80 | ± | 4.15 |  | 188.03 | ± | 25.14 |  |
| 28ºC 8 g/L |  | 3.57 | ± | 0.69 |  | 77.84 | ± | 15.46 |  | 13.33 | ± | 2.76 |  | 44.41 | ± | 8.04 |  |
| 28ºC 9 g/L |  | 11.81 | ± | 2.29 |  | 100.71 | ± | 14.87 |  | 18.63 | ± | 2.92 |  | 70.90 | ± | 6.86 |  |
| 28ºC 10 g/L |  | 2.56 | ± | 0.49 |  | 66.82 | ± | 12.03 |  | 17.30 | ± | 3.24 |  | 64.43 | ± | 11.23 |  |

**Table 14.** Endogenous total cytokinin pools based on structural and functional forms (pmol g^-1^ DW) in somatic embryos from nine different treatments (maturated at 18, 23 and 28ªC and at 8, 9 and 10gL^-1^ Gelrite®) mean ± standard error. Nucleotides and N-glucosides were under the limit of detection.

| **Treatment** |  | **Bases** | | |  | **Ribosides** | | |  | **O-glucosides** | | |
| --- | --- | --- | --- | --- | --- | --- | --- | --- | --- | --- | --- | --- |
| 18ºC 8 g/L |  | 154.52 | ± | 19.03 |  | 203.38 | ± | 39.03 |  | 0.67 | ± | 0.11 |
| 18ºC 9 g/L |  | 10.70 | ± | 0.58 |  | 74.39 | ± | 8.92 |  | 0.22 | ± | 0.09 |
| 18ºC 10 g/L |  | 12.06 | ± | 1.07 |  | 131.27 | ± | 16.74 |  | 0.47 | ± | 0.07 |
| 23ºC 8 g/L |  | 166.03 | ± | 28.93 |  | 164.63 | ± | 20.84 |  | 0.47 | ± | 0.14 |
| 23ºC 9 g/L |  | 30.39 | ± | 4.20 |  | 105.98 | ± | 16.24 |  | 0.19 | ± | 0.04 |
| 23ºC 10 g/L |  | 21.65 | ± | 3.89 |  | 285.66 | ± | 42.27 |  | 0.27 | ± | 0.06 |
| 28ºC 8 g/L |  | 16.52 | ± | 1.64 |  | 122.12 | ± | 25.30 |  | 0.51 | ± | 0.12 |
| 28ºC 9 g/L |  | 26.93 | ± | 4.93 |  | 174.21 | ± | 20.81 |  | 0.91 | ± | 0.02 |
| 28ºC 10 g/L |  | 49.27 | ± | 6.11 |  | 98.02 | ± | 16.70 |  | 3.82 | ± | 0.81 |

**Fig. 1.** *Ex vitro* survival (%) in *Pinus radiata* plantlets coming from embryogenic

cell lines maturated at three different temperatures (18, 23 and 28ºC) and three gellan

gum concentrations (8, 9, 10 g L^-1^).

**
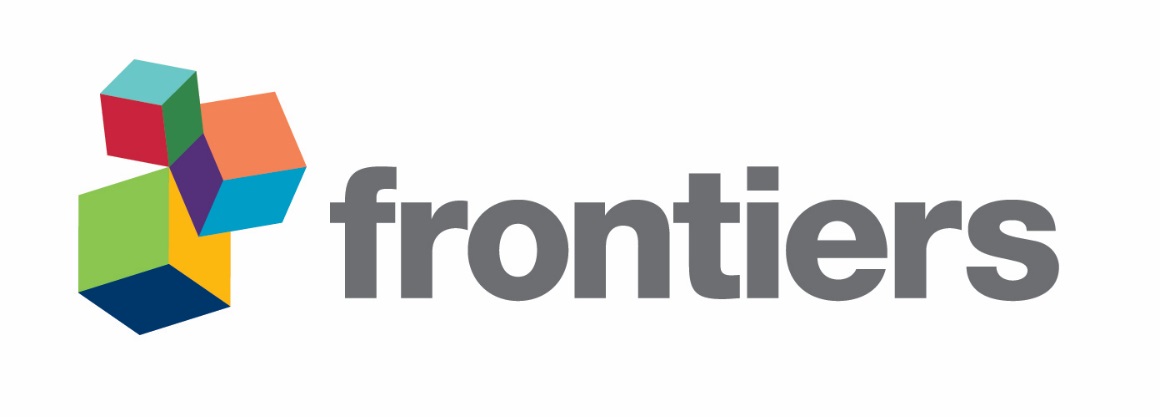
**
